# Clinical results of jugular-axillary approach for extracorporeal left ventricular assist device

**DOI:** 10.3389/fcvm.2026.1735102

**Published:** 2026-08-26

**Authors:** Shilin Wang, Qianwen Liu, Ping Li, Changdong Zhang, Wenqian Wu, Hao Liu, Peiwen Yang, Lingyun Fang, Wei Su, Jamshid H. Karimov, Nianguo Dong, Shu Chen

**Affiliations:** 1 Department of Cardiovascular Surgery, Union Hospital, Tongji Medical College, Huazhong University of Science and Technology, Wuhan, China; 2 Department of Technology, Boea Wisdom (Hangzhou) Network Technology Co., Ltd., Hangzhou, China; 3 Department of Ultrasound Medicine, Union Hospital, Tongji Medical College, Huazhong University of Science and Technology, Wuhan, China; 4 Hubei Province Key Laboratory of Molecular Imaging, Wuhan, China; 5 Department of Vascular Surgery, Traditional Chinese and Western Medicine Hospital of Wuhan, Tongji Medical College, Huazhong University of Science and Technology, Wuhan, China; 6 Research Collaboration Group, Cleveland, OH, United States; 7 Cardiovascular Innovation and Research, Cleveland, OH, United States

**Keywords:** axillary artery, extracorporeal, left ventricular assist device, percutaneous left ventricular assist device, right jugular vein, surgical access

## Abstract

**Objectives:**

The traditional approach of placing the percutaneous extracorporeal left ventricular assist device (LVAD) through the femoral artery and vein has several downsides, primarily restricting patients’ movement and requiring a specific type of vein drainage cannula. To address this clinical limitation, we proposed a technique of placing the Extra-LVAD through the right axillary artery and jugular vein to bypass these issues.

**Methods:**

Five patients underwent extra-LVAD implantation through the right axillary artery and jugular vein approach at Wuhan Union Hospital between May 2022 and January 2023. In this retrospective study, we compared echocardiographic parameters and dynamic lab data before and after LVAD support.

**Results:**

All patients successfully bridged heart transplants with no major or minor adverse events, and no patient required an oxygenator during a median support duration of 10.0(±5.40) days. Hemodynamic support was effective, as evidenced by improved right ventricular function (tricuspid annular plane systolic excursion increased from 1.7 ± 0.2 to 1.9 ± 0.2; right ventricular area change rate from 33.2 ± 3.7% to 39.0 ± 8.0%) and stable end-organ perfusion markers (e.g., lactate and creatinine trends) maintained over 6 days post-implantation. Key physiological indicators, including mean arterial pressure, central venous pressure, renal and hepatic function markers remained stable or demonstrated improving trends over the 6-day post-implantation period. Post-transplant, 4 patients achieved a left ventricular ejection fraction above 60% without mechanical circulatory support, although one patient required an intra-aortic balloon pump.

**Conclusions:**

In our 5-patient group, percutaneous insertion of an extra-LVAD through the right axillary artery and jugular vein may be viable and effective. This could be a favorable access point for future procedures. However, large samples and patient outcomes in different centers still need to be studied.

## 1 Introduction

The extracorporeal left ventricular assist device (Extra-LVAD) is commonly used as a temporary support for patients with severe heart failure, serving as a bridge to heart transplantation (1–3). However, the conventional femoral artery (FA) and vein approach has some limitations. It forces patients to bed rest, limiting their mobility and complicating nursing care, and increases the risk of lower limb ischemia (4–7). Besides, some centers lack the vein drainage cannula long enough to span the distance from the femoral vein to the left atrium (LA) (8), challenging the establishment of a stable loop in LA.

We have developed a new approach to address these challenges using the right jugular vein and axillary artery (AA) for percutaneous placement. This technique may improve patient mobility, not necessitate a specific catheter type, and reduce the risk of ischemia. Our study showed the viability and effectiveness of this technique based on a retrospective review of five patients.

## 2 Materials and methods

### 2.1 Study population

This study was conducted under a protocol approved by the Institutional Review Board of Union Hospital, Affiliated with Tongji Medical College, Huazhong University of Science and Technology, Wuhan, China (number: 2023-0429, approval date: June 6, 2023). The study protocol conforms to the ethical guidelines of the 1975 Declaration of Helsinki. From May 2022 to January 2023, five patients at our center underwent Extra-LVAD placement via the right jugular vein and AA. These patients were selected based on their height, all of them were above 164 cm. Their baseline demographic and clinical characteristics are presented in Table 1. We performed a retrospective analysis of data for these patients, which includes echocardiographic parameters and laboratory data indices, both before and after Extra-LVAD support.

**Table 1:** Baseline patient demographic characteristics.

Patient ID	#1	#2	#3	#4	#5	Average/frequency
Age (yrs)	24	56	56	48	61	49.0 (±13.2)
Sex	Male	Male	Male	Male	Male	Male (100.0%)
Etiology	Dilated cardiomyopathy	Ischemic	Dilated cardiomyopathy	Valvular	Hypertrophic cardiomyopathy	1 Ischemic (20.0%) 2 Dilated cardiomyopathy (40.0%) 1 Valvular (20.0%) 1 Hypertrophic Cardiomyopathy (20.0%)
Functional capacity						
NYHA class Ⅲb or Ⅳ	Y	Y	Y	Y	Y	5 (100.0%)
INTERMACS class 7	Y	Y	Y	Y	Y	5 (100.0%)
Physical examination						
Weight (kg)	78	55	68	81	59	68.2 (±10.2)
Height (cm)	185	165	164	185	170	173.8 (±9.4)
BMI (kg/cm^2^)	22.79	20.2	25.28	23.67	20.42	22.5 (±1.9)
Systolic blood pressure (mmHg)	92	95	83	90	87	89.4 (±4.1)
Diastolic blood pressure (mmHg)	51	58	52	60	44	53.0 (±5.7)
Medical history						
Diabetes mellitus	N	Y	Y	N	Y	3 (60.0%)
Hypertension	N	N	N	N	N	0 (0%)
Hyperlipidemia	Y	N	Y	N	N	2 (40.0%)
Chronic kidney disease	N	N	Y	Y	Y	3 (60.0%)
Previous CVA/TIA	N	N	N	N	N	0 (0)
Peripheral vascular disease	N	N	N	N	Y	1 (20.0%)
Current or former smoker	N	Y	N	N	N	1 (20.0%)
Selected laboratory values						
BUN (mmol/L)	5.23	13.57	5.23	8.64	13.95	9.3 (±3.8)
Creatinine (*μ*mol/L)	119.1	88.1	119.1	141.4	160.4	125.6 (±24.3)
Echocardiography						
LVEF (%)	22.8	35	22.8	15.8	15	22.3 (±7.2)
LVEDd (cm)	8	8.7	8	11.9	7.2	8.8 (±1.6)
Duration of support (days)	13	8	19	4	6	10.0(±5.40)

Values are mean ± SD or *n* (%).

NYHA, New York Heart Association; INTERMACS, interagency registry for mechanically assisted circulatory support; BMI, body mass index; CVA, cerebrovascular accident; TIA, transient ischemic attacks; BUN, blood urea nitrogen; LVEF, left ventricular ejection fraction; LVEDd, left ventricular end diastolic dimension; Y, yes; N, no.

### 2.2 Percutaneous placement technique

An 8 mm- vascular graft (Terumo Corp, Tokyo, Japan) was stitched end-to-side to the axillary artery. Then the prosthetic vessel was connected to the perfusion tube of the MoyoAssist Extra-VAD device (magAssist Co, Suzhou, China) using an 8–10 mm connector.

**Figure 1:**
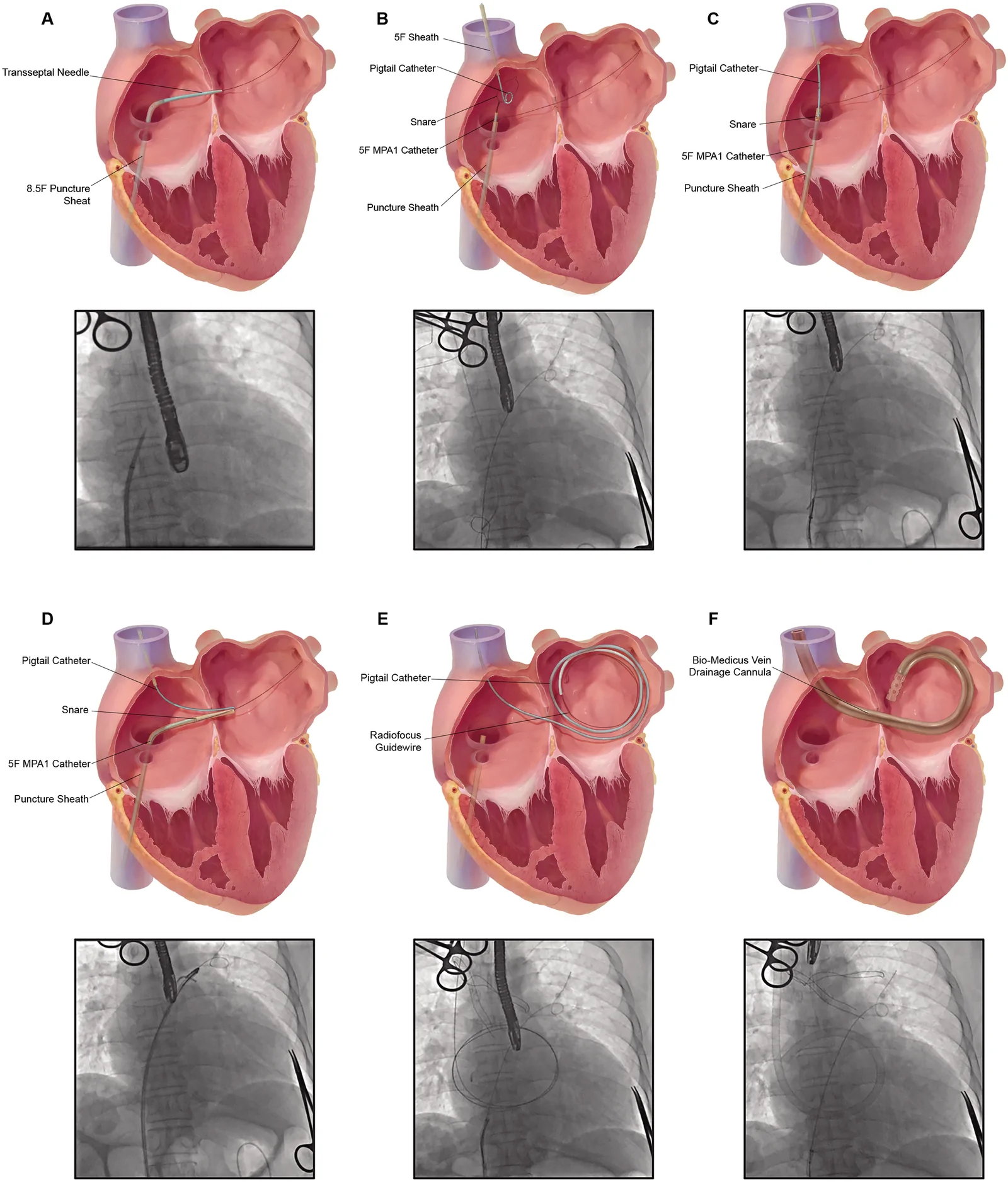
Illustration of the procedure for inserting a vein drainage cannula for extra-LVAD through the right jugular vein. (**A**) Atrial septal puncture was conducted. (**B**) A snare secured the tip of the pigtail catheter in the right atrium or superior vena cava. (**C**) The pigtail catheter was pulled into the puncture sheath. (**D**) The puncture sheath was advanced into the left atrium. (**E**) The pigtail catheter formed two large loops through the Radiofocus guidewire. (**F**) A Bio-Medicus vein drainage cannula was positioned and a loop is formed.

Atrial septal puncture was performed through the femoral vein using an 8.5 F puncture sheath and a transseptal needle (Abbott Vascular, Menlo Park, CA, USA). A 260-cm rigid guidewire (Cordis, Santa Clara, CA, USA) was then advanced into the left superior pulmonary vein (LSPV) (Figure 1A). The sheath was pulled back to the orifice of the inferior vena cava while the guidewire remained in the LSPV. An 8 mm*4 cm non-compliant balloon (Cook Medical, Bloomington, IN) expanded the septal puncture site.

Under ultrasound guidance, a 6 F sheath was inserted into the right internal jugular vein. A 5 F MPA1 catheter (Cordis, Santa Clara, CA, USA) and a snare (Starway, Beijing, China) were delivered to the inferior vena cava through the puncture sheath in the femoral vein (F-MPA1). A pigtail catheter was advanced to the superior vena cava through the 5 F sheath in the right internal jugular vein. After securely capturing the tip of the pigtail catheter (Figure 1B), the MPA1 catheter was retracted into the puncture sheath, pulling the pigtail catheter into it as well (Figure 1C). The puncture sheath was then advanced into the LA along the rigid guidewire, pulling the pigtail catheter into the LA (Figure 1D). The snare was then released, leaving the pigtail catheter free in the LA. A Radiofocus guidewire (Terumo, Tokyo, Japan) was advanced into the LA, forming 3 large loops. The pigtail catheter was then advanced into the LA to form 2 large loops through the Radiofocus guidewire (Terumo Corporation, Tokyo, Japan), as shown in Figure 1E. The Radiofocus guidewire was then swapped with a 260 cm rigid guidewire (Cordis, Santa Clara, CA, USA), forming 2 large loops in the LA through the pigtail catheter. After removing the pigtail catheter and 5 F sheath in the jugular vein, a 19 or 21 F Bio-Medicus venous drainage cannula (Medtronic, Minneapolis, MN) was advanced into the LA along the 260 cm rigid guidewire. The cannula formed a loop in the LA (Figure 1F). It was then connected to the inflow cannula of the Extra-VAD device.

**Figure 2:**
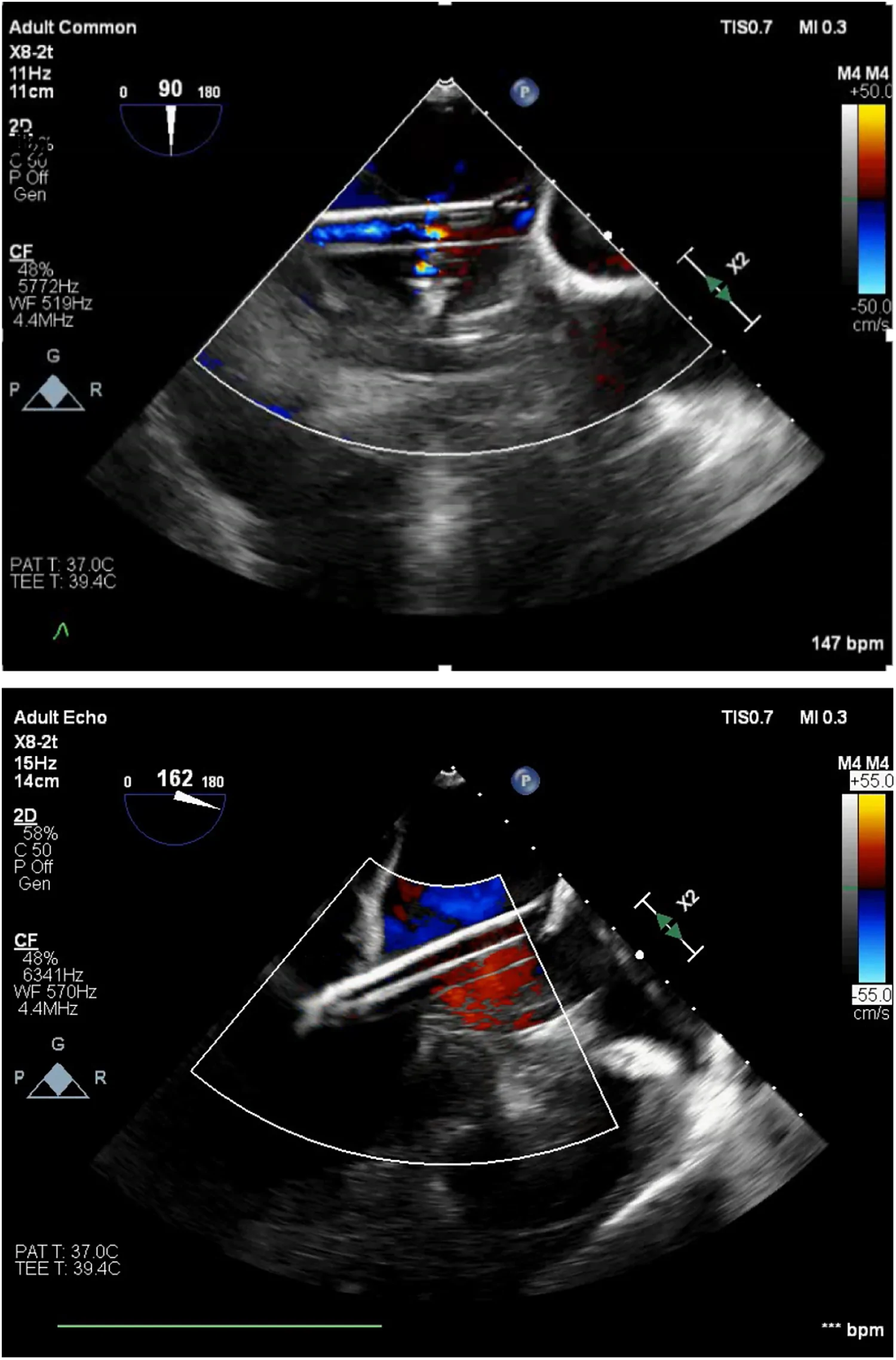
Transesophageal echocardiography after an LVAD insertion.

**Figure 3:**
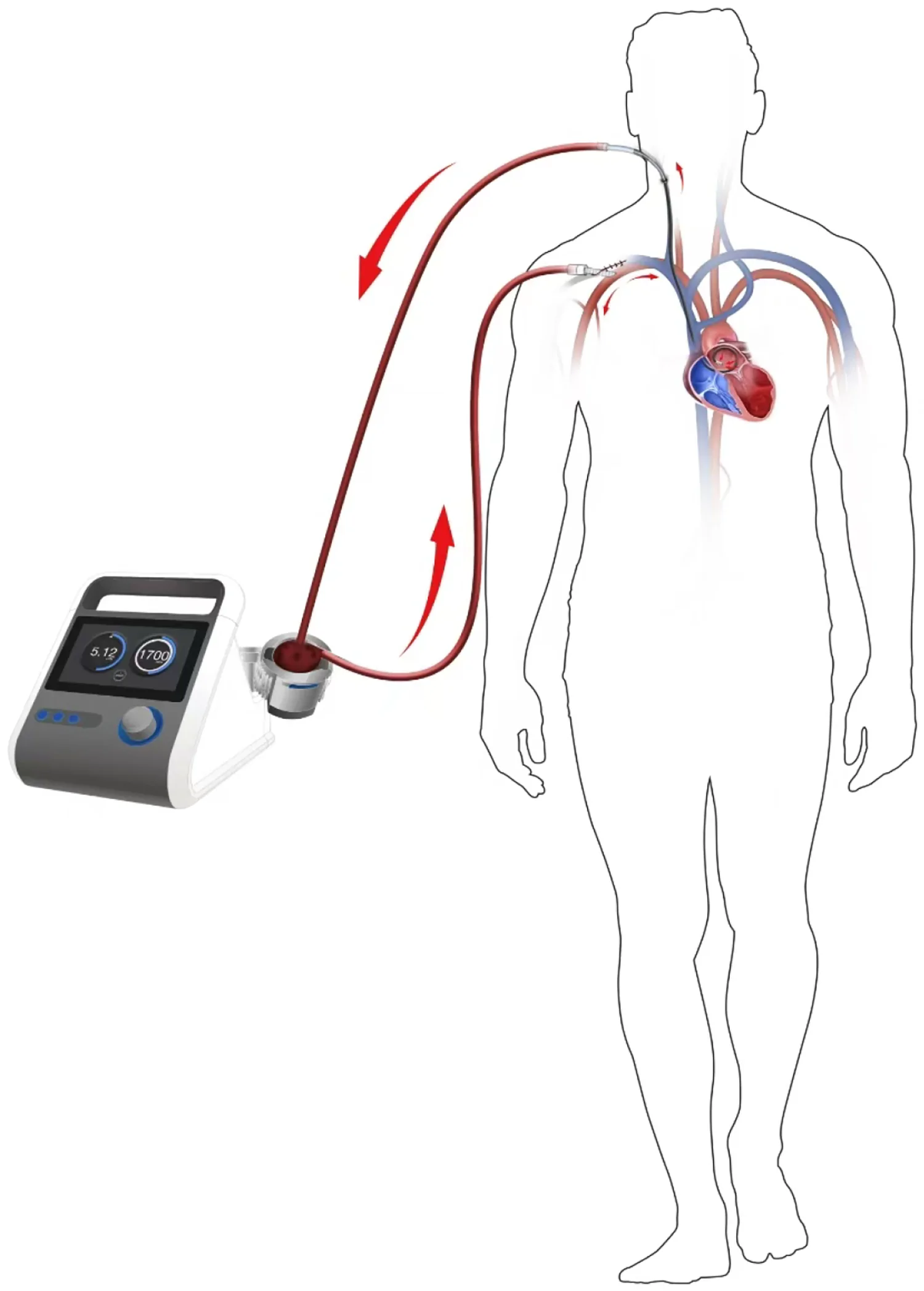
Patient's condition after an LVAD insertion.

Transesophageal echocardiography was utilized to confirm the location of the vein drainage cannula, as depicted in Figure 2. The final intubation site of LVAD is depicted in Figure 3.

### 2.3 LVAD management

We adhered to our systemic heparinization protocol, aiming for an activated clotting time (ACT) between 180 and 200, and used the MoyoAssist® Extra-VAD (magAssist Co. Inc., Suzhou, China) (9) as the LVAD pump, establishing the LA-AA system after venting. The pump flow rate was initially set at an average of 2.5 ± 0.8 liter per min (LPM), with a speed of 2,223.2 ± 240.5 revolutions per minute (RPM), and adjustments were made to maintain the mean arterial pressure above 60 mmHg. All patients stayed in the cardiovascular surgery department's intensive care unit and were allowed mobility. Since all patients had a normal lung function, we did not use oxygenators.

### 2.4 Clinical outcome measures

Successful implantation was defined as the successful placement of the Extra-LVAD without termination of the procedure. Data from hemodynamic evaluations and selected laboratory tests were compared before and after Extra-LVAD support.

### 2.5 Definitions of adverse events

Major complications were defined as issues related to the Extra-LVAD during its implantation and while it was in use. These complications included significant bleeding, ischemic events, extracorporeal LVAD malfunction, and catheter malposition. Significant bleeding is defined as requiring surgery or ≥2 units of red cells. Ischemic issues mainly involved neurological events like cerebrovascular accidents and transient ischemic attacks (TIA), major limb ischemia, and mesenteric ischemia due to thrombosis, embolism, and compromised blood flow. Catheter malposition was identified through daily bedside transthoracic echocardiography and x-ray.

Minor complications were those tied to the Extra-LVAD that weren't classified as major but required additional intervention. These encompassed LVAD-related infections, brachial plexus injuries, and catheter-related problems such as hematomas or aneurysms. Infectious complications were recognized as cases of bacteremia during LVAD support that demanded systemic antibiotics. Arterial duplex ultrasounds diagnosed issues like catheter-related hematomas and aneurysms.

### 2.6 Statistical analysis

Mean values ± standard deviation were used for continuous data and percentages for categorical data. Given the small sample size and the heterogeneity of baseline patient characteristics, the data in this study are presented by descriptive statistics.

## 3 Results

### 3.1 Study population

Five patients selected based on their height were included in the study, with the average height of 173.8 (±9.4) cm. However, the effective length of the venous drainage tubes available in Chinese market was less than 50 cm. Through the femoral vein, after puncturing the atrial septum, the short venous drainage tubes could not reach the left pulmonary veins, or some of the side holes on the tubes would stay in the right atrium causing drainage to non-oxygenated blood. Therefore, they underwent Extra-LVAD placement via the right jugular vein and axillary artery. The average operation time was 209.0 (±64.1) min. Table 1. outlines the participants’ basic demographic and clinical details. For privacy protection, the five enrolled patients were designated by study codes #1 through #5. All the patients were male, averaging 49.0 (±13.2) years of age, and all exhibited advanced heart failure symptoms, classified as NYHA class IV and INTERMACS class 7. Despite intensive medication, their condition remained critical. The average duration of Extra-LVAD support was 10 (±5.4, median 6, from 4 to 19) days, serving as a bridge to heart transplants.

### 3.2 Clinical efficacy

**Figure 4:**
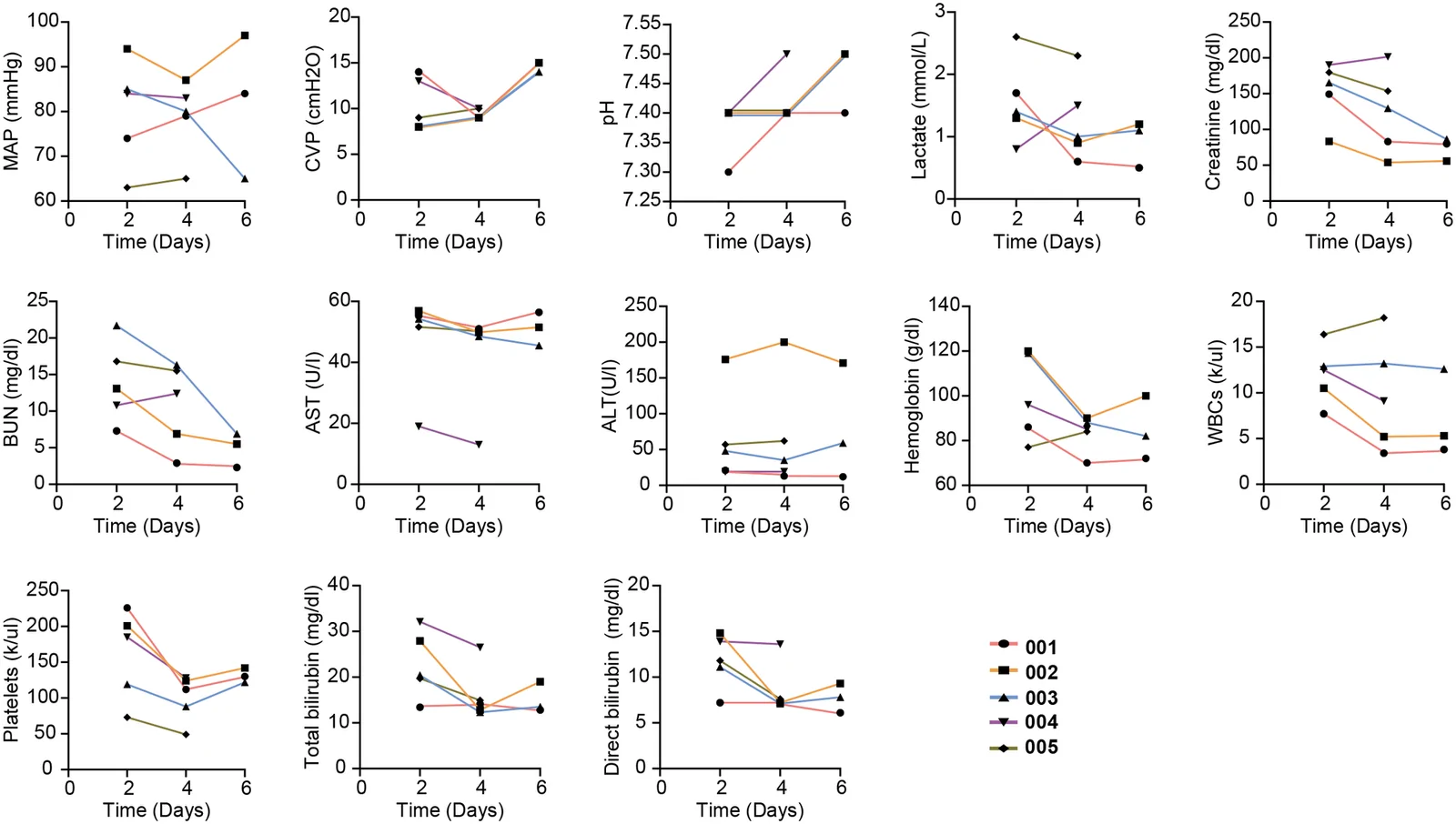
Dynamic changes of key physiological indicators during extra-LVAD implantation.

The insertion of the Extra-LVAD through the right axillary artery and jugular vein was successful in all 5 patients, with no complications arising from the implant. No patients received oxygenator during the period of waiting for transplantation. All patients could sit upright or ambulate during support, and everyone engaged in nurse-guided walking. Table 2 compares pertinent hemodynamic values and coagulation indicators before and after Extra-LVAD support. The average value of tricuspid annular plane systolic excursion changed from 1.7 ± 0.2 to 1.9 ± 0.2, and the right ventricular area change rate changed from 33.2 ± 3.7 to 39.0 ± 8.0, which reflect right ventricular function. The pump flow rate, speed, and dynamic changes in key physiological indicators during Extra-LVAD implantation are shown in Table 3. Figure 4 shows the line chart of key physiological indicators, including mean arterial pressure, central venous pressure, haematological indexes, and markers reflecting end-organ perfusion. These key physiological indicators are collected on the 2nd, 4th and 6th days after the implantation of Extra-LVAD.

**Table 2:** Comparison of pertinent hemodynamic values and coagulation indicators before and after extra-LVAD support.

Parameter	Patient no.	Before implantation	After implantation
LVEF (%)	#1	22.8	25
	#2	35	25
	#3	23	22
	#4	15.8	15
	#5	15	11
	Average	22.3 (±7.2)	19.6 (±5.6)
TAPSE (cm)	#1	1.7	1.9
	#2	1.6	1.9
	#3	1.9	2.1
	#4	1.4	1.5
	#5	1.9	2.2
	Average	1.7 (±0.2)	1.9 (±0.2)
RVFAC (%)	#1	28.2	30.8
	#2	37	46
	#3	38	49
	#4	32.3	29
	#5	30.6	40
	Average	33.2 (±3.7)	39.0 (±8.0)
Pt (s)	#1	13.1	13.6
	#2	13.9	18.8
	#3	14.2	13.8
	#4	19.2	14.2
	#5	13.5	15.3
	Average	14.8 (±2.2)	15.1 (±1.9)
INR	#1	1.1	1.05
	#2	1.18	1.77
	#3	1.22	1.18
	#4	1.63	1.12
	#5	1.05	1.21
	Average	1.2 (±0.2)	1.3 (±0.3)

Values are mean ± SD.

TAPSE, tricuspid annular plane systolic excursion; RVFAC, right ventricular area change rate; Pt, prothrombin time; INR, international normalized ratio; other abbreviations as in Table 1.

Before Implantation: Day 0.

After Implantation: Average days of LVAD support: 6.2 (±6.1); median: 3 days; range from 2 to 18 days.

**Table 3:** The pump flow rate, speed, and dynamic changes in key physiological indicators during extra-LVAD implantation.

Parameter	Time* (Day)	#1	#2	#3	#4	#5
Flow rate (LPM)	0	2.57	1.82	1.57	3.7	2.97
Speed (RPM)	0	2,549	2,000	2,261	2,400	1,906
MAP (mmHg)	2nd	74	94	85	84	63
	4th	79	87	80	83	65
	6th	84	97	65	—	—
CVP (cmH_2_O)	2nd	14	8	8	13	9
	4th	9	9	9	10	10
	6th	15	15	14	—	—
pH	2nd	7.3	7.4	7.4	7.4	7.4
	4th	7.4	7.4	7.4	7.5	7.4
	6th	7.4	7.5	7.5	—	—
Lactate (mmol/L)	2nd	1.7	1.3	1.4	0.8	2.6
	4th	0.6	0.9	1	1.5	2.3
	6th	0.5	1.2	1.1	—	—
Creatinine (μmol/L)	2nd	149.3	83.4	165.3	190	179.7
	4th	83.1	53.8	129.5	201.5	153.6
	6th	79.8	56	86.4	—	—
BUN (mmol/L)	2nd	7.3	13.1	21.7	10.8	16.8
	4th	2.9	6.9	16.3	12.4	15.5
	6th	2.3	5.5	6.9	—	—
AST (U/I)	2nd	55.3	56.9	54.3	19	51.6
	4th	51	49.9	48.5	13	50.2
	6th	56.4	51.5	45.5	—	—
ALT(U/I)	2nd	21	176	48	19	57
	4th	13	200	35	19	62
	6th	12	171	59	—	—
Hemoglobin (g/dl)	2nd	86	120	119	96	77
	4th	70	90	88	85	84
	6th	72	100	82	—	—
WBCs (k/uL)	2nd	7.7	10.5	12.9	12.5	16.4
	4th	3.4	5.2	13.2	9.1	18.2
	6th	3.8	5.3	12.6	—	—
Platelets (k/uL)	2nd	226	201	119	185	73
	4th	112	124	88	128	49
	6th	130	142	122	—	—
Total bilirubin (μmol/L)	2nd	13.4	27.9	20.4	32.1	19.7
	4th	14.1	12.8	12.3	26.5	14.9
	6th	12.8	19	13.5	—	—
Direct bilirubin (μmol/L)	2nd	7.2	14.8	11.1	13.9	11.8
	4th	7.2	7.2	7.1	13.6	7.6
	6th	6.1	9.3	7.8	—	—

LPM, liters per minute; RPM, revolutions per minute; MAP, mean arterial pressure; CVP, central venous pressure; WBCs, white blood cells; AST, aspartate aminotransferase; ALT, alanine aminotransferase; other abbreviations as in Tables 1 and 2.

* The time is counted from Extra-LVAD was implanted.

All patients successfully bridged to heart transplantation. After transplant, no patient received extracorporeal membrane oxygenation, while patient 4 received intra-aortic balloon pump. Except patient 4, the LVEF of all other patients was above 60%. Among them, two patients developed complications. Patient 2 experienced arrhythmia on the 19th day and died as a result, and pulmonary aspergillosis was found in patient 5. Transplantation outcomes among Extra-LVAD supported patients is shown in Table 4.

**Table 4:** Transplantation outcomes among extra-LVAD supported patients.

Parameter	#1	#2	#3	#4	#5
Primary graft dysfunction					
LVEF (%) on Day 7*	72	65	65	60	65
LVEF (%) on Day 14*	66	71	70	58	65
Using IABP	N	N	N	Y	N
Using MCS devices	N	N	N	N	N
Complications after transplantation	N	Arrhythmia and death	N	N	Pulmonary aspergillosis

* Patients underwent echocardiography on the 7th and 14th days after heart transplantation.

IABP, intra-aortic balloon pump; MCS, mechanical circulatory support; other abbreviations as in Table 1.

### 3.3 Adverse events

All 5 patients successfully underwent the implantation procedure and progressed to subsequent treatments. No major or minor adverse events were observed during the implantation or Extra-LVAD support. No neurological events including ischemic stroke, hemorrhagic stroke, and TIA were observed as well. No internal jugular artery thrombosis was observed when tubes removing in transplantation procedure. However, 2 patients reported right arm mild numbness after implantation lasted for 2–3 days, but there was no swelling. The symptoms did not affect the patients’ lives, and there's no residual symptoms occurred after the removal of the Extra-LVAD.

## 4 Discussion

The Extra-LVAD is a device designed to aid patients experiencing escalating severity of heart failure, even while on optimal medical therapy. Conventionally, this device is implanted through the femoral vein and artery (6), an approach is known to have drawbacks, such as restricting mobility (4–7), needing special vein drainage cannulas, potentially increasing the risk of lower limb ischemia. Our study observed five patients in whom the Extra-LVAD was successfully positioned via the right jugular vein and axillary. With this approach, patients can move more freely, making nursing care easier, and vein drainage cannulas of different sizes can also be used. Besides, this approach also works for patients with certain vascular diseases and reduces the risk of infection from groin incisions. Furthermore, the left atrial drainage tube was inserted by a fully interventional approach, without the need for open-chest surgery. Compared with previous studies (10), it causes less trauma. All of these advantages make the procedure more flexible and widely used.

The artificial graft was directly sutured to the axillary artery without the use of intraoperative distal perfusion cannulation, primarily due to the high mobility of the axillary region. Unlike the femoral artery, which possesses a longer straight segment and remains relatively stable regardless of lower extremity movement, the axillary artery is subject to significant positional changes. Placing a distal perfusion cannula in this area could therefore result in vessel injury from cannula tip movement. Additionally, direct cannulation was also avoided owing to concerns about potential right upper extremity ischemia.

All the patients in this study had normal lung function and no oxygenator was added to the Extra-LVAD system. With the transjugular venous approach, the venous drainage cannula is sufficiently long to form a complete loop within the left atrium. This configuration ensures positional stability, effectively eliminating the risk of inadvertent left atrial cannula dislodgement that could cause rapid systemic desaturation, which is frequently encountered with the femoral vein approach. Therefore, the likelihood of desaturation due to cannula dislodgement is substantially reduced. Besides, the oxygenator is also an important contributing factor to platelet consumption, which could be one of multiple perioperative factors contributing to thrombocytopenia. Furthermore, the limited durability of the oxygenator necessitates replacement, which introduces additional procedural risks. For patients with normal lung function who only require left ventricular support, these risks can be reasonably avoided by omitting the oxygenator. Therefore, no oxygenators were add routinely to the patients.

During the subsequent transplantation process, we routinely place a separate drainage catheter directly into the superior vena cava via a thoracic approach after sternotomy, without the need for additional auxiliary tubing. Should Swan-Ganz monitoring be required, it could be inserted via the subclavian or left internal jugular vein. The superior vena cava offers sufficient luminal diameter to accommodate such placement without obstruction, given the appropriate positioning.

The primary limitation of our study is its retrospective nature, based on a single-center's experience with a small sample size. We didn't find any statistical difference in right heart function, possibly due to the small sample size and short duration of LVAD support. We recorded no major or minor adverse events during the study. Lack of magnetic resonance imaging and detailed hemodynamic data also hindered our ability to compare peripheral circulation directly. And our study was conducted exclusively in an Asian cohort. The relatively smaller body habitus (lower body mass index and body surface area) and the lower baseline incidence of venous thromboembolism in Asian populations may limit the generalizability of our findings to Western patients, particularly given the higher rates of thrombosis following percutaneous ventricular assist device placement reported in North America. We are fully aware that expanding our success rate more widely may require more procedural experience and a larger sample size in multi-ethnic and larger-scale patient populations. Although the procedure may be easy to use, variations in procedural experience could pose an increased risk. Nevertheless, due to its advantages, this minimally invasive method may still present a new avenue for applying the extra-LVAD.

## 5 Conclusions

In the 5-patient group, our retrospective study suggests that the jugular-axillary approach for Extra-LVAD may enhance patient mobility and minimize some complications, such as lower limb ischemia and infection of groin incisions. Eliminating the need for a specific type of vein drainage cannula may also broaden the use of extracorporeal LVAD. More studies using larger patient groups, including additional clinical centers, could provide more detailed information on the proposed technique.

## Data Availability

The original contributions presented in the study are included in the article/Supplementary Material, further inquiries can be directed to the corresponding author.
